# UCP2 promotes NSCLC proliferation and glycolysis via the mTOR/HIF‐1α signaling

**DOI:** 10.1002/cam4.6938

**Published:** 2024-01-12

**Authors:** Cailu Song, Qing Liu, Jing Qin, Lingrui Liu, Zhigang Zhou, Han Yang

**Affiliations:** ^1^ State Key Laboratory of Oncology in South China, Guangdong Provincial Clinical Research Center for Cancer Sun Yat‐sen University Cancer Center Guangzhou China; ^2^ Changde Hospital, Xiangya School of Medicine Central South University (The First People's Hospital Of Changde City) Changde China

**Keywords:** glycolysis, HIF‐1α, mTOR, NSCLC, proliferation, UCP2

## Abstract

**Background:**

Metabolic disturbance is a hallmark of cancers. Targeting key metabolic pathways and metabolism‐related molecular could be a potential therapeutic approach. Uncoupling protein 2 (UCP2) plays a pivotal part in the malignancy of cancer and its capacity to develop resistance to pharmaceutical interventions. However, it is unclear about the mechanism of how UCP2 acts in the tumor growth and metabolic reprogramming process in non‐small cell lung cancer (NSCLC).

**Methods:**

Here, we conducted qRT‐PCR to investigate the expression of UCP2 in both NSCLC tissues and cell lines. Subsequent functional studies including colony formation assay, CCK‐8 assay, and glycolysis assay were conducted to investigate the functions of UCP2 in NSCLC. The regulatory mechanism of UCP2 toward the mammalian target of rapamycin (mTOR) and hypoxia‐inducible factor‐1 alpha (HIF‐1α) signaling in NSCLC was confirmed through western blotting.

**Results:**

We observed a significant upregulation of UCP2 in both NSCLC tissues and cell lines. The increased expression of UCP2 has a strong association with a worse outlook. Silencing UCP2 remarkably dampened NSCLC cell proliferation and glycolysis capacities. Mechanically, UCP2 promoted NSCLC tumorigenesis partially via regulating the mTOR/HIF‐1α axis.

**Conclusion:**

Taken together, we explored the functions as well as the mechanisms of the UCP2/mTOR/HIF‐1α axis in NSCLC progression, uncovering potential biological signatures and targets for NSCLC treatment.

## 1 INTRODUCTION

Non‐small cell lung cancer (NSCLC) is recognized as an aggressive and deadly malignancy globally. Recently, there has been remarkable revolutionary progress made in NSCLC management, especially the progress in precision medicine and targeting therapy.^1^, ^2^ Though the advances in management of NSCLC have significantly improved the screening, diagnosis, and treatment of NSCLC, there are still cases where the outlook for NSCLC patients remains unfavorable.^3^, ^4^

Metabolism reprogramming, as a hallmark of cancer, is vital for cancer cells to sustain uncontrolled growth and survival under stressful conditions.^5^ In NSCLC, glucose and mitochondrial metabolism play vital roles in tumorigenesis, that metabolism reprogramming leads to enhanced cell growth and proliferation.^6^ Therefore, targeting specific molecular that correlated with cancer cell metabolic reprogramming is a promising therapeutic strategy.^7^

Uncoupling protein 2 (UCP2) has been found upregulated in various cancer types, leading to tumorigenesis, cancer progression and chemotherapy resistance.^8^ Recently, studies have shown that UCP2 could induce a metabolic shift toward the glycolytic pathway, sustaining the Warburg effect which promotes cancer progression.^9^ Increasing data has indicated that UCP2 is associated with energy metabolism regulation in cancers. Through metabolic and energetic transformation, oxidative stress and reactive oxygen species (ROS) can be decreased by metabolic and energetic transformation, and overexpression of UCP2 in cancer cells to avoid cell apoptosis. Thus, UCP2 represents a promising target for therapeutic interventions in cancer treatment. Nevertheless, there is still limited knowledge regarding the specific functions and underlying mechanisms of UCP2 in NSCLC.

Here, the expression of UCP2 in NSCLC tissues and cell lines were detected. We observed a significant upregulation of UCP2 in NSCLC tissues versus neighboring nonmalignant tissues, and UCP2 was overexpression in cell lines as well. Functionally, inhibition of UCP2 suppressed NSCLC cell proliferation and glycolysis. Mechanically, UCP2 promoted NSCLC progression by regulating the signaling pathway involving the mammalian target of rapamycin (mTOR) and hypoxia‐inducible factor‐1 alpha (HIF‐1α). As a result, the potential of UCP2 as both a diagnostic biomarker and a therapeutic target for addressing NSCLC holds great promise.

## 2 METHODS

### 2.1 Data collection

We downloaded UCP2 expression profile in lung cancer from the TCGA lung cancer cohort in the UCSC Xena project, including 483 tumor tissues as well as 347 normal tissues. We used Kaplan–Meier Plotter website to generate the overall survival (OS) curve as well as the progression free survival (PFS) curve of UCP2 in lung cancer.

### 2.2 Clinical sample collection

Forty paired fresh NSCLC tissue samples and corresponding para‐carcinoma nonmalignant tissue samples were collected at Sun Yat‐sen University Cancer Center. To ensure the integrity of the tissue, we instantly stored all the tissue samples in liquid nitrogen. Total RNA was isolated and conducted to qRT‐PCR analysis. The research protocols involving human samples were permitted by the Ethics Committee of Sun Yat‐sen University Cancer Center and conducted in compliance with the Declaration of Helsinki. All patients were informed and signed consent forms.

### 2.3 Cell culture and transfection

Normal lung cells (Beas2b cells) and NSCLC cells (PC9, H1299, H1975 and A549 cells) were purchased from ATCC (USA). Cell authenticity was confirmed by DNA fingerprinting. Assays were routinely performed to detect mycoplasma infection.

The Lipofectamine 3000 system (Invitrogen, USA) was used to perform transfection. UCP2 siRNAs were supplied by Ruibo (China). Here are the siRNA sequences: si‐UCP2#1, 5′‐CACTGTCGACGCCTACAAGACCATC‐3′; si‐UCP2#2, 5′‐GTCATAGGTCACCAGCTCAGCACAG‐3′; si‐UCP2#3, 5′‐GACGAGAUACAUGAACUCUGC‐3′.

### 2.4 qRT‐PCR analysis

TRIzol (Invitrogen) was employed to isolate total RNA. Takara PrimeScript™ RT reagent Kit and TB Green Premix Ex Taq™ (Japan) were applied for qRT‐PCR assays. The expressions of mRNAs were detected by the 2^−ΔΔCt^ method. β‐Actin was used as the control for mRNA expression. The primers sequences for qRT‐PCR were supplied by Ruibo: β‐actin, Forward, 5′‐CGGGAAATCGTGCGTGAC‐3′, Reverse, 5′‐CAGGAAGGAAGGCTGGAAG‐3′; UCP2, Forward, 5′‐ TCCTGAAAGCCAACCTCATG‐3′, Reverse, 5′‐GGCAGAGTTCATGTATCTCGTC‐3′.

### 2.5 Cell counting kit‐8 assay (CCK‐8)

After transfection, 10^3^ cells were seeded in 96‐well plates, and then, the cells underwent incubation under proper conditions for 48 h. We added 10 μL CCK‐8 solution (Dojindo, Japan) to the culture medium and underwent incubation for 2 h. The OD values were detected at 450 nM on a Bio‐Tek EPOCH2 microtiter plate reader (USA), and the data were recorded.

### 2.6 Colony formation assay

After transfection, 10^3^ cells were seeded in 6‐well plates. Then the cells were incubated at 37°C for 14 days until visible clones were observed with the naked eye. Later, using paraformaldehyde we fixed the clones and stained it with crystal violet. Finally, we captured and counted the clones.

### 2.7 Detection of glycolytic metabolism

Glucose Assay Kit (Beyotime, China) was employed to detected the glucose consumption in NSCLC cells. After transfection, cells were seeded (10^6^ cells/well). We collected the cell culture medium to evaluate the reduction of glucose concentration. On the other hand, Lactate Assay Kit (Dojindo, Japan) was utilized to measure the lactate production in cell culture medium. Finally, ADP/ATP Ratio Assay Kit (Dojindo) was employed to measure the ATP/ADP ratio.

### 2.8 Western blotting

Briefly, PMSF and RIPA lysis buffer (Beyotime) were employed to extract the NSCLC cell proteins. To detect the concentration of cell proteins, Pierce BCA Protein assay kit was conducted. Cell proteins were isolated by 10% SDS‐PAGE before transferring to PVDF membranes. Using 5% skim milk, we blocked PVDF membranes at room temperature for 1 h. Later, with primary antibodies, the membranes were incubated overnight at 4°C, including UCP2 (1:1000, #DF8626, Affinity, USA), mTOR (1:500, #AF6308, Affinity), p‐mTOR(Ser2448) (1:500, #AF3308, Affinity), S6K (1:500, #AF6226, Affinity), p‐S6K(Thr389) (1:500, #AF3228, Affinity), 4E‐BP (1:500, #AF6432, Affinity), p‐4E‐BP(Thr70) (1:500, #AF2308, Affinity), HIF‐1α (1:500, #AF1009, Affinity) and α‐Tubulin (1:1000, #AF4651, Affinity). Next day, with HRP‐linked secondary antibody (1:3000, #S0001, Affinity), the membranes were washed before incubation at room temperature for 2 h. Finally, the membranes were visualized by ECL Detection Reagent (Yeasen, China), and ImageJ software was used to quantify the relative grayscale value.

### 2.9 Statistical analyses

We used SPSS 25.0 software to conduct statistical analyses. Comparation of the differences between groups was conduct with t‐tests. The data were expressed as the mean ± standard deviation (SD). Statistical significance was determined for differences with a *p* < 0.05.

## 3 RESULTS

### 3.1 The upregulation of UCP2 in lung cancer and correlated with a poor prognosis

**FIGURE 1:**
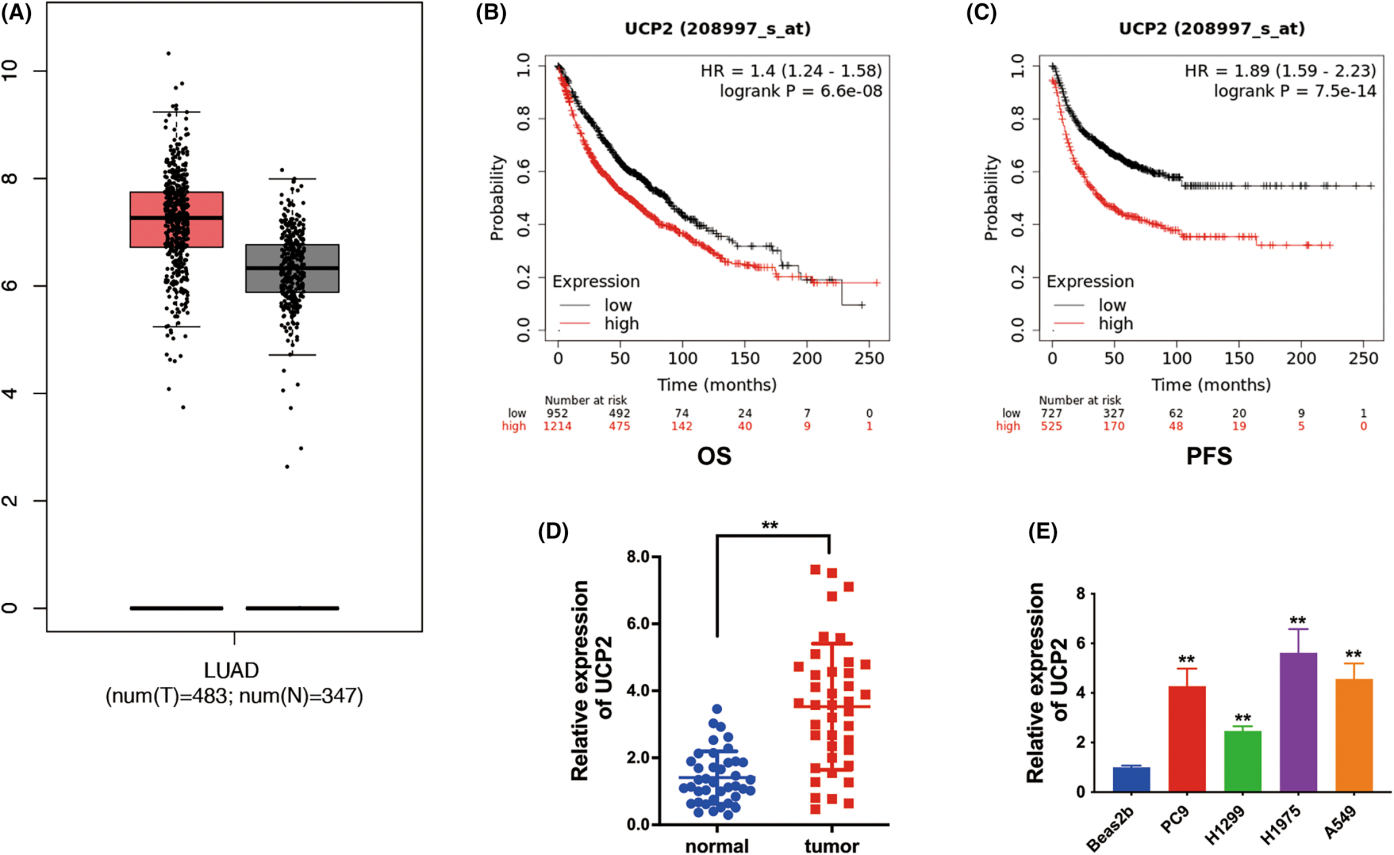
The upregulation of uncoupling protein 2 (UCP2) in lung cancer and correlated with a poor prognosis. (A) UCP2 expression profiles in lung tissue, including tumors and normal tissues from the TCGA database. (B) The overall survival (OS) curves of patients with lung cancer in different UCP2 expression levels from the Kaplan–Meier plotter website. (C) The progression free survival (PFS) curves of patients with lung cancer in different UCP2 expression levels from the Kaplan–Meier Plotter website. (D) UCP2 expression in 40 non‐small cell lung cancer (NSCLC) and paired normal tissues. (E) The UCP2 expression in NSCLC cell lines **p < 0.01.

We downloaded the microarray series data from the TCGA database to determine the expression of UCP2 in lung cancer. The result showed that UCP2 was increased in lung cancer tissues (Figure 1A). Next, we investigate the effects of UCP2 high expression on patients' outcome with lung cancers through Kaplan–Meier Plotter database. We discovered that high expression level of UCP2 in lung cancer led to worse OS (Figure 1B) and PFS (Figure 1C). Then, we assessed UCP2 expression in 40 pairs of NSCLC tissues and corresponding para‐carcinoma nonmalignant tissue samples. The results demonstrated that UCP2 was upregulated in NSCLC tissues (Figure 1D). Further experiments showed that UCP2 also expressed at high levels in NSCLC cell lines (Figure 1E).

### 3.2 NSCLC proliferation were suppressed by UCP2 inhibition

**FIGURE 2:**
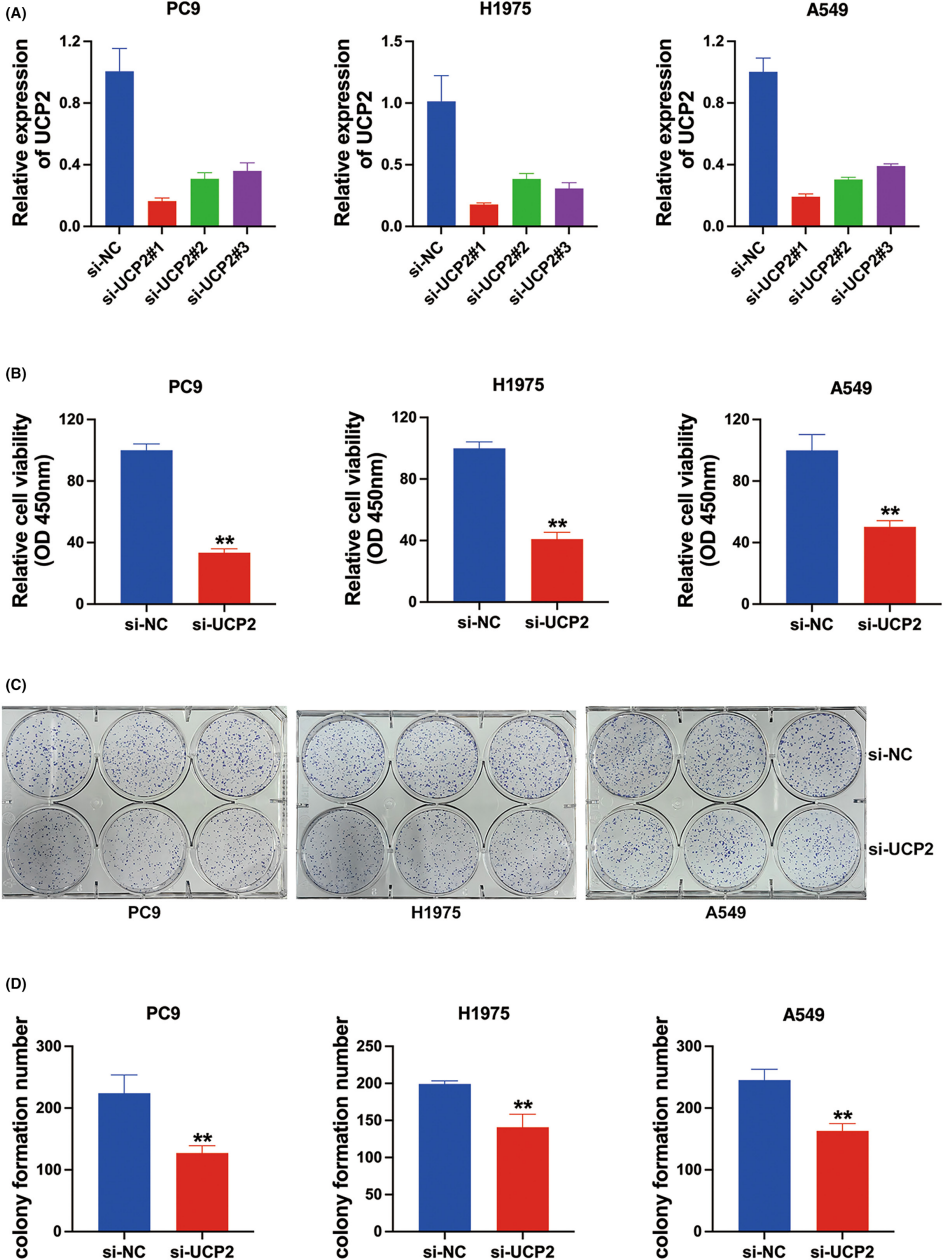
Non‐small cell lung cancer (NSCLC) proliferation were suppressed by uncoupling protein 2 (UCP2) inhibition. (A) qRT‐PCR was used to confirm the effect of si‐RNAs in PC9, H1975, and A549 cell lines. (B) CCK‐8 assay showed the effect of downregulation of UCP2 on cell growth. (C) Colony formation analysis show the role of downregulation of UCP2 on cell proliferation. (D) Statistic graph of colonies **p < 0.01.

We designed three siRNAs in PC9, H1975, and A549 cell lines to decrease the UCP2 expression based on the overexpression of UCP2 in NSCLC. Figure 2A shows that the UCP2 expression were significantly downregulated after si‐UCP2#1 transfection. Therefore, we selected si‐UCP2#1 to perform the following experiments. In CCK‐8 assays, downregulation of UCP2 suppressed NSCLC cell growth (Figure 2B). Moreover, inhibition of UCP2 reduced NSCLC cell colony formation number (Figure 2C,D). In general, downregulation of UCP2 inhibited cell proliferation in NSCLC.

### 3.3 UCP2 inhibition suppressed cell glycolysis in NSCLC

**FIGURE 3:**
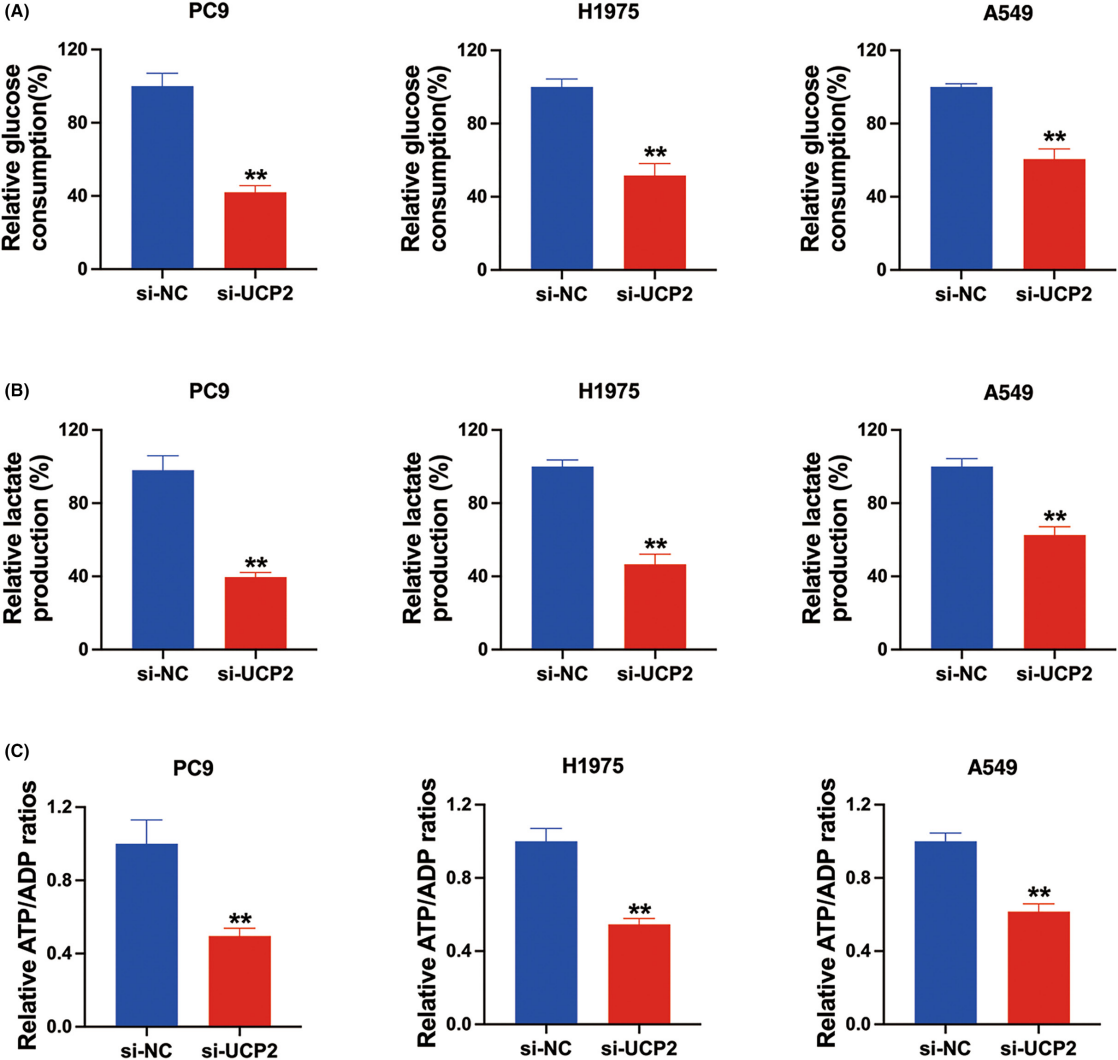
Uncoupling protein 2 (UCP2) inhibition suppressed cell glycolysis in non‐small cell lung cancer (NSCLC). (A) Glucose consumption levels were detected to reflect the glycolytic metabolism in NSCLC cells. (B) Lactate production levels were detected to reflect the glycolytic metabolism in NSCLC cells. (C) ATP/ADP ratio were detected to reflect the glycolytic metabolism in NSCLC cells **p < 0.01.

Next, glucose consumption analysis, lactate production analysis as well as ATP/ADP ratio analysis were conducted in PC9, H1975, and A549 cell lines to detect the impacts of UCP2 on NSCLC glycolysis. We observed that knockdown of UCP2 considerably decreased glucose consumption levels (Figure 3A), lactate generation levels (Figure 3B), and the ATP/ADP ratio (Figure 3C) in NSCLC. These findings indicated that knockdown of UCP2 could inhibit NSCLC glycolysis.

### 3.4 UCP2 promoted NSCLC progression through the mTOR/HIF‐1α pathway

**FIGURE 4:**
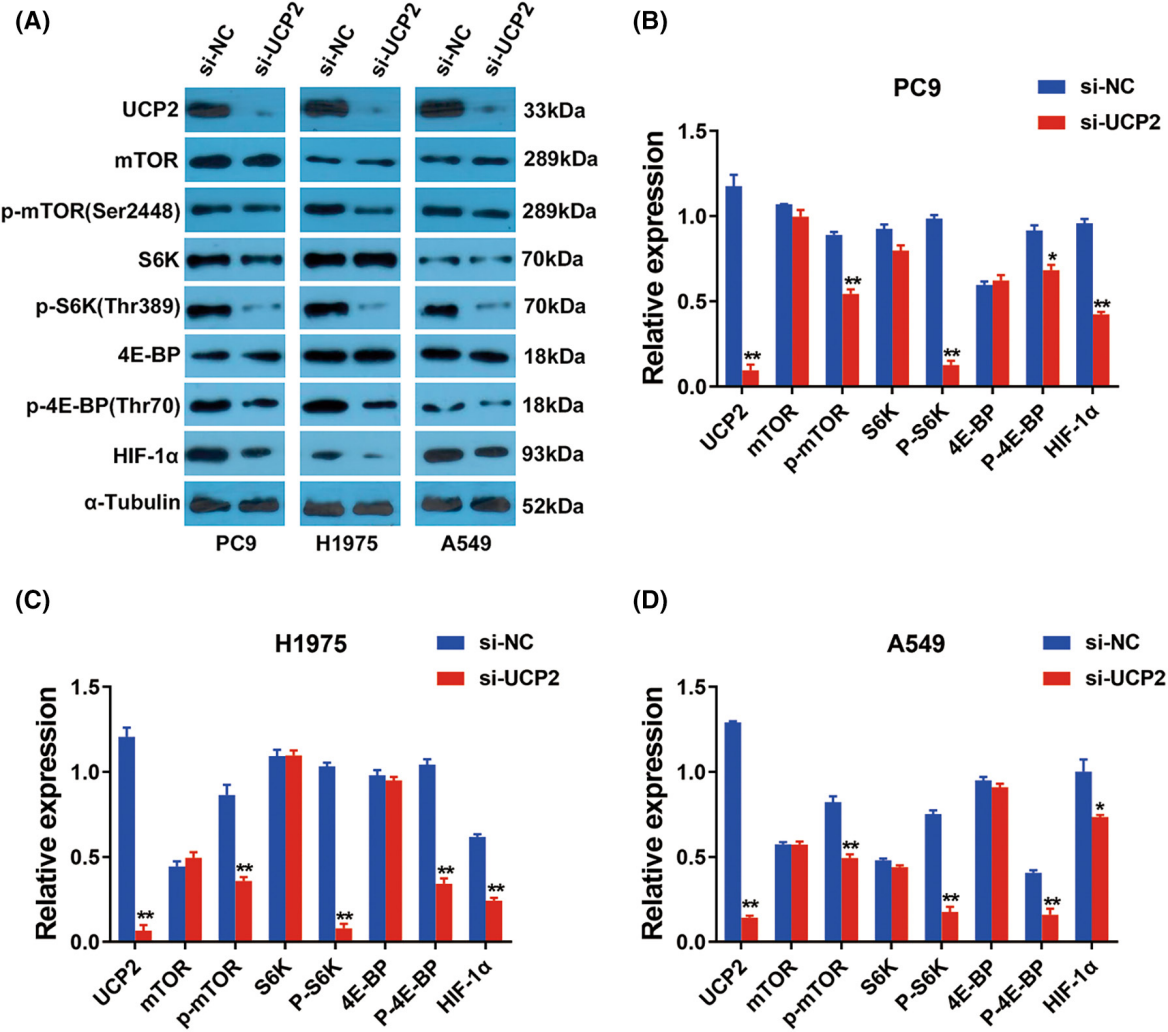
UCP2 promoted NSCLC progression through the mTOR/HIF‐1α pathway. (A) Western blotting assay underwent conduct after PC9, H1975, and A549 cell lines were transfected. (B) ImageJ software was used to quantify the protein expression. **p < 0.01.

Finally, we explored the mechanism of UCP2 in NSCLC advancement. Previous research shows that UCP2 promotes tumorigenesis via enhancing the Akt/mTOR pathway in melanoma.^10^ Besides, HIF‐1α activation plays a pivotal part in in regulating tumor progression. And agents that suppress the activation of mTOR could also negatively affect the expression of HIF‐1α.^11^ However, the impacts of UCP2 on mTOR/HIF‐1α signaling have not yet been explored in NSCLC. Thus, we investigated whether silencing UCP2 suppressed the mTOR/HIF‐1α signaling. Western blotting showed UCP2 inhibition notably suppressed the phosphorylation of mTOR, whereas the overall expression of mTOR remained unaffected (Figure 4). Moreover, Inhibition of UCP2 suppressed the phosphorylation of ribosomal S6 kinase (S6K) and 4E‐binding protein (4E‐BP), which represented the critical downstream targets of the mTOR signaling (Figure 4). The phosphorylation of S6K and 4E‐BP could modulate cell proliferation and protein synthesis, including the HIF‐1α proteins. And UCP2 suppression notably decreased HIF‐1α expression in NSCLC cell lines, which represented the downstream component of the mTOR/S6K/4E‐BP signaling (Figure 4). To summarize, the above findings revealed that UCP2 promoted NSCLC tumorigenesis partly via the mTOR/S6K/4E‐BP/HIF‐1α pathway.

## 4 DISCUSSION

NSCLC accounts for the most cases of lung cancers. The past decade has witnessed the significant advances in NSCLC management, which have increased patient outcome and quality of life. However, recurrence and metastasis importantly drawback the outcome of some NSCLC patients.^12^, ^13^ The OS rate for late‐stage NSCLC remains poor. Further investigation is required regarding the specific treatment targets and proper combinations of these targeted drugs.^14^, ^15^

In NSCLC, the genotype polymorphism of UCP2 is correlated with risk for NSCLC susceptibility.^16^ Here, using data from the TCGA database, in lung cancer samples, we observed the UCP2 expression was increased. Besides, in both NSCLC tissues and cell lines, we also confirmed that UCP2 was upregulated. Moreover, upregulation of UCP2 was associated with unfavorable outcome in lung cancer patients (Figure 1), indicating that UCP2 could act as potential diagnostic biomarker for NSCLC.

Studies showed that overexpression of UCP2 significantly increases cell viability, proliferation and mitochondrial respiration, and is correlated with reduced OS.^17^ Here, we found that inhibition of UCP2 suppressed NSCLC cell proliferation (Figure 2), and NSCLC cell glycolysis (Figure 3). However, the underlying mechanism of how UCP2 functions in the tumor growth and metabolic reprogramming process in NSCLC remains largely unknown.

Metabolic disturbance is a hallmark of cancers. Cancer cells sustain increased energetic demands via metabolic reprogramming to promote cancer progression and treatment resistance has been reported.^18^ In NSCLC, inhibition of tumor glycolysis could suppress cell survival and proliferation.^19^ Thus, targeting key metabolic pathways and metabolism‐related molecular could be a potential therapeutic approach.^20^

UCP2 is increased in NSCLC and is positively associated with hypoxia markers' expression, such as HIF‐1α, acting as a vital player in NSCLC hypoxia response network.^21^ UCP2 plays a vital role in cancer aggressiveness and drug resistance from its capacity to suppress ROS production and inhibit cell apoptosis. UCP2 inhibitor genipin can suppress growth of pancreatic adenocarcinoma cells and trigger cell apoptosis via promoting GAPDH nuclear translocation.^22^ Inhibiting the function of UCP2 by genipin results in enhanced ROS production and reduced cell survival in NSCLC cells.^23^ Therefore, the mechanism of UCP2 in regulating NSCLC cell growth and metabolism is worth further investigation.

The mTOR pathway is heavily involved in tumorigenesis and progression in various cancers.^24^, ^25^ Inhibiting mTOR signaling pathway could suppress EMT in NSCLC cells, supporting mTOR as a promising treatment focus for NSCLC.^26^ Recently, a variety of targeted inhibitors against mTOR are under a series of clinical trials in NSCLC.^27^ It is reported that through the AKT/mTOR signaling, UCP2 could associate with DDX5 to regulate the metabolic plasticity in NSCLC.^28^ Moreover, the administration of genipin, inhibiting UCP2, could considerably downregulated the expression of Akt and mTOR.^29^ In pancreatic adenocarcinoma, the combination of UCP2 inhibitor genipin and mTOR inhibitor everolimus results in synergistic suppression of cancer cell growth and induction of cell apoptosis.^22^ P‐mTOR is a direct indicator of mTOR signaling pathway activity. The level of p‐mTOR reflects the activity level of the mTOR signaling pathway. mTOR complex 1 (mTORC1) is one of the functional complexes of mTOR, which is involved in cell proliferation, metabolism and protein synthesis regulation. After activation, mTORC1 could phosphorylate downstream proteins, such as S6K and 4E‐BP, which represented the critical downstream targets of the mTOR signaling. Here, we found that inhibition of UCP2 notably suppressed the phosphorylation of mTOR, S6K and 4E‐BP, whereas the overall expression of mTOR, S6K and 4E‐BP remains unaffected (Figure 4).

Studies show that mTOR signaling has a critical impact on regulating the transcription and translation of HIF‐1α.^30^ The phosphorylation of S6K and 4E‐BP could modulate the protein synthesis of HIF‐1α. Hypoxia and HIF‐1α could lead to intrinsic or acquired resistance towards anticancer drugs.^31^ Via inducing glycolysis to increase energy production in cancer cells, HIF‐1α could prevent cancer cells from senescence and promote cancer cells proliferation. Inhibiting the HIF‐1α pathway could suppress NSCLC progression via enhancing cell cycle arrest and accelerating cellular senescence.^32^ Therefore, suppressing HIF‐1α is a potential option for NSCLC therapies. In this study, we revealed that knockdown of UCP2 notably suppressed HIF‐1α expression in NSCLC cells, which represented the downstream component of the mTOR/S6K/4E‐BP signaling (Figure 4). Taken together, these finding revealed that UCP2 promoted NSCLC tumorigenesis partly through the mTOR/S6K/4E‐BP/HIF‐1α pathway.

## 5 CONCLUSION

Altogether, the above results indicated the underlying mechanisms of UCP2 promoting NSCLC cell proliferation and glucose metabolism partly via the mTOR/HIF‐1α signaling. The exploration of targeted therapeutics aimed at inhibiting glycolytic metabolism, either as standalone treatments or in conjunction with existing therapies, holds significant promise as a therapeutic strategy for NSCLC. Additional investigations are necessary to assess the impacts of combination inhibition of UCP2 and mTOR/HIF‐1α by targeted drugs, which may provide a novel treatment strategy for NSCLC.

## AUTHOR CONTRIBUTIONS

**Cailu Song:** Formal analysis (equal). **Qing Liu:** Data curation (equal). **Jing Qin:** Formal analysis (equal). **Lingrui Liu:** Data curation (equal). **Zhigang Zhou:** Data curation (equal). **Han Yang:** Conceptualization (lead).

## FUNDING INFORMATION

This work received financial support from by the Science and Technology Program of Guangzhou (Grant Number 202102080084).

## CONFLICT OF INTEREST STATEMENT

The authors state that they haven't conflicts of interest to disclose regarding the present study.

## ETHICAL APPROVAL

All processes involving human subjects were conducted according to the Ethics Committee of Sun Yat‐sen University Cancer Center (2022–270‐01) and the Declaration of Helsinki, which was revised in 2013. All the patients involved were informed and signed consent forms.

## Data Availability

Data sharing is not applicable to this article as no new data were created or analyzed in this study.
